# Supplementary material for: Structure of a new capsid form and comparison with A-, B-, and C-capsids clarify herpesvirus assembly
Source: J Virol. 2025 Jul 3;99(7):e00504-25. doi: 10.1128/jvi.00504-25 (PMC12282113; doi:10.1128/jvi.00504-25)
Supplement: Supplemental material — Fig. S1 to S3; Table S1. [file jvi.00504-25-s0001.docx]

**Supplemental information**

**Structure of a new capsid form and comparison with A-, B- and C-capsids clarify herpesvirus assembly**

Alexander Stevens^a,b,c,d,*^, Saarang Kashyap^a,b,*^, Ethan Crofut^a,b,*^, Ana Lucia Alverez-Cabrera^a,b^, Jonathan Jih^a,b,c^, Yun-Tao Liu^a^, Z. Hong Zhou^a,b,c,d^

^a^Department of Microbiology, Immunology, and Molecular Genetics, University of California, Los Angeles (UCLA), Los Angeles, CA, USA

^b^California NanoSystems Institute, UCLA, Los Angeles, CA, USA

^c^Molecular Biology Institute, UCLA, Los Angeles, CA, USA

^d^Department of Chemistry and Biochemistry, UCLA, Los Angeles, CA, USA

*Contributed equally, order determined by drawing straws

**#Address correspondence to Z. Hong Zhou,** [**hong.zhou@ucla.edu**](mailto:hong.zhou@ucla.edu) **(phone 310-694-7527)**

**Figure S1. CryoEM image processing workflow for portal vertex reconstructions.**

**Figure S2. D-capsids occur in a spectrum of genome occupancies.**

**Figure S3. Cross section of D-capsid portal vertex without terminal genome.**

**Table S1. CryoEM data collection.**

**Movie S1. 3DFlex volume series of the A-capsid portal vertex.**

**Movie S2. 3DFlex volume series of the A-capsid floor at the portal vertex.**

**Movie S3. 3DFlex volume series of the B-capsid portal vertex.**

**Movie S4. 3DFlex volume series of the B-capsid floor at the portal vertex.**

**Movie S5. 3DFlex volume series of the C-capsid portal vertex.**

**Movie S6. 3DFlex volume series of the C-capsid floor at the portal vertex.**

**Movie S7. 3DFlex volume series of the D-capsid portal vertex.**

**Movie S8. 3DFlex volume series of the D-capsid floor at the portal vertex.**


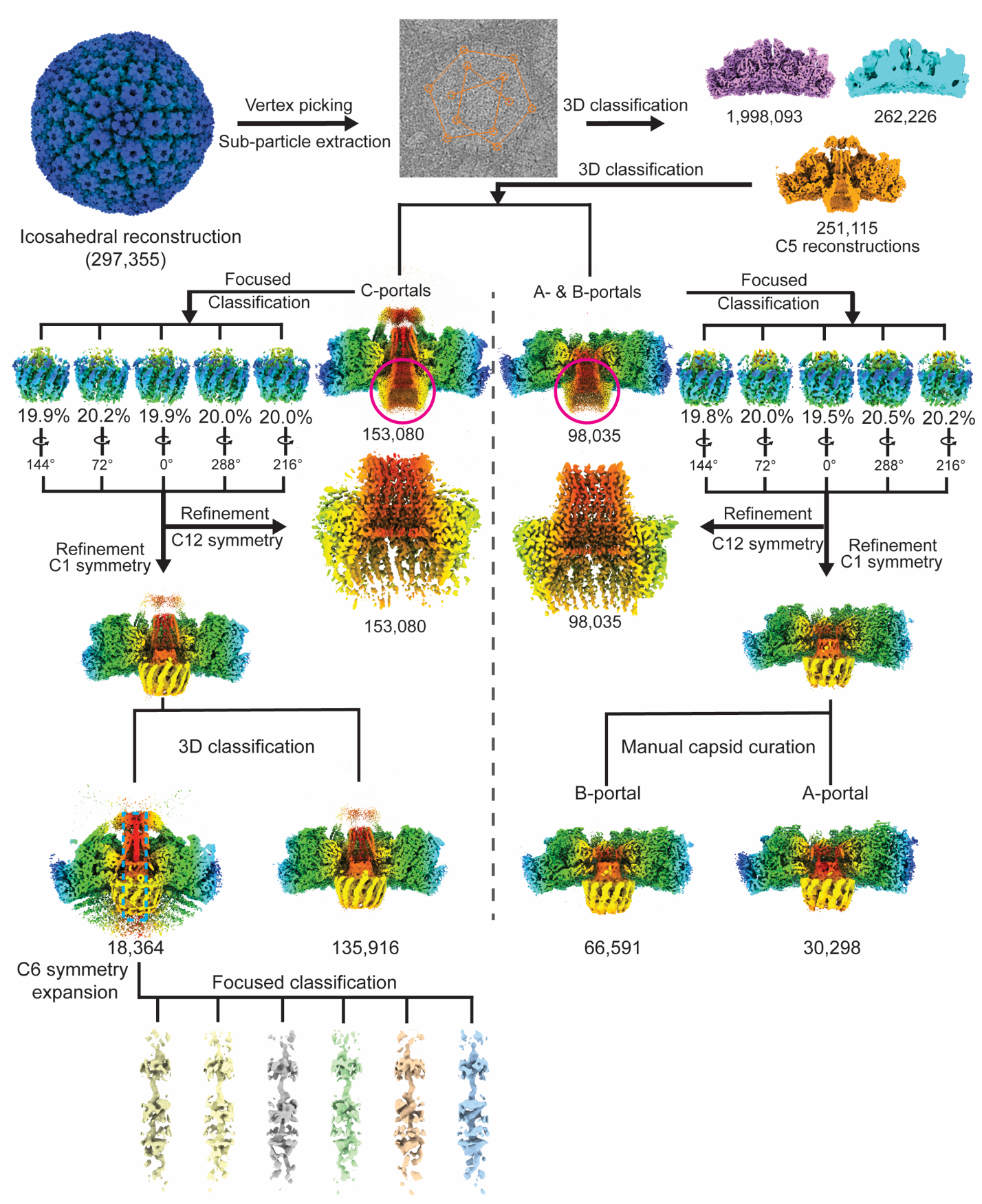


**Figure S1: CryoEM image processing workflow for portal vertex reconstructions.**


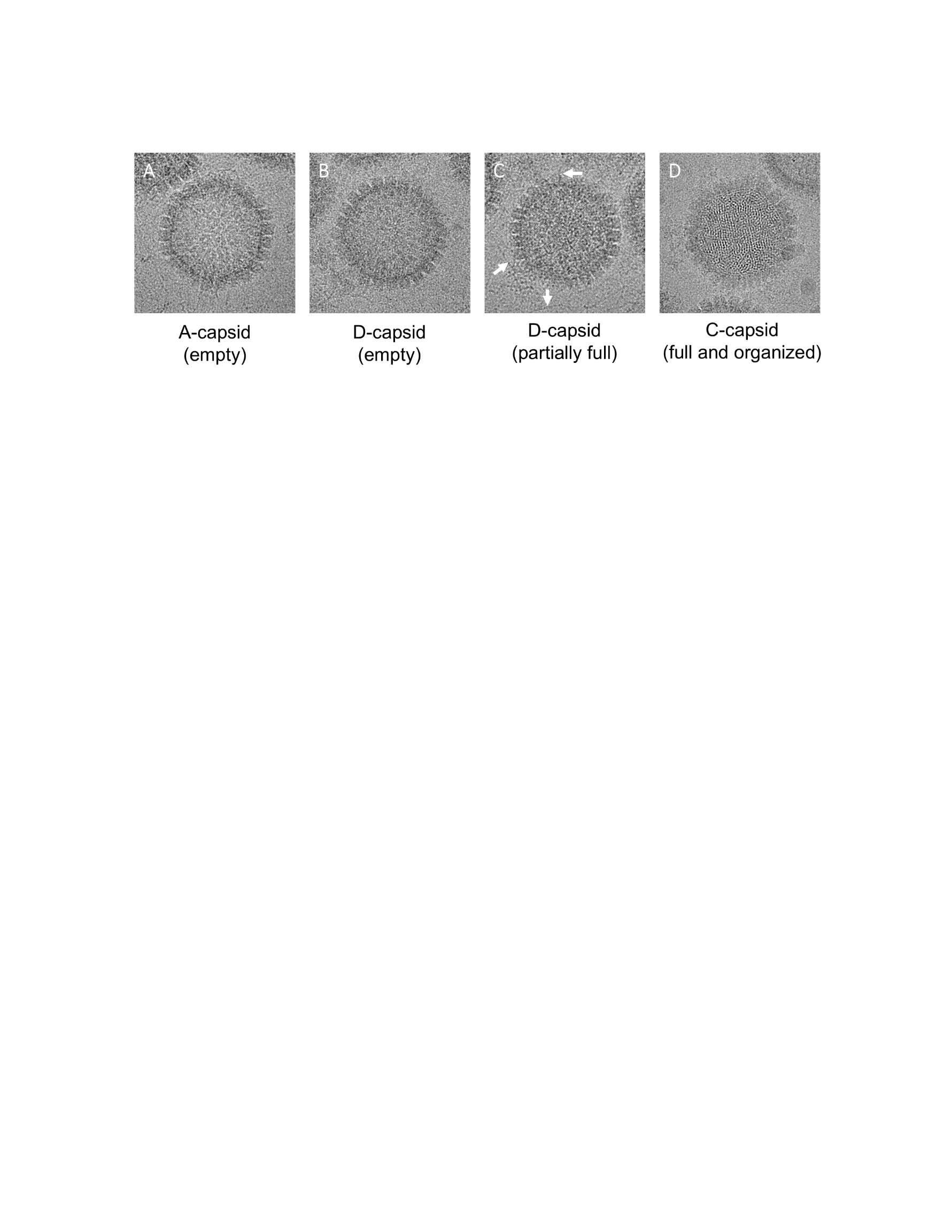


**Figure S2: D-capsids occur in a spectrum of genome occupancies.**

(A) Typical A-capsid micrograph, appearing as an empty capsid. (B) D-capsid micrograph appearing empty, like an A-capsid. (D) Another D-capsid micrograph, appearing filled but not clearly organized. D-capsids occur as a continuum of capsid occupancies, with some empty and some partially filled, but lack the clear genome organization of C-capsids. White arrows indicate free nucleic acid strands, which are common in the sample and may be due to genome ejection in D-capsids. (D) Typical C-capsid micrograph, displaying clear organization of the genome.


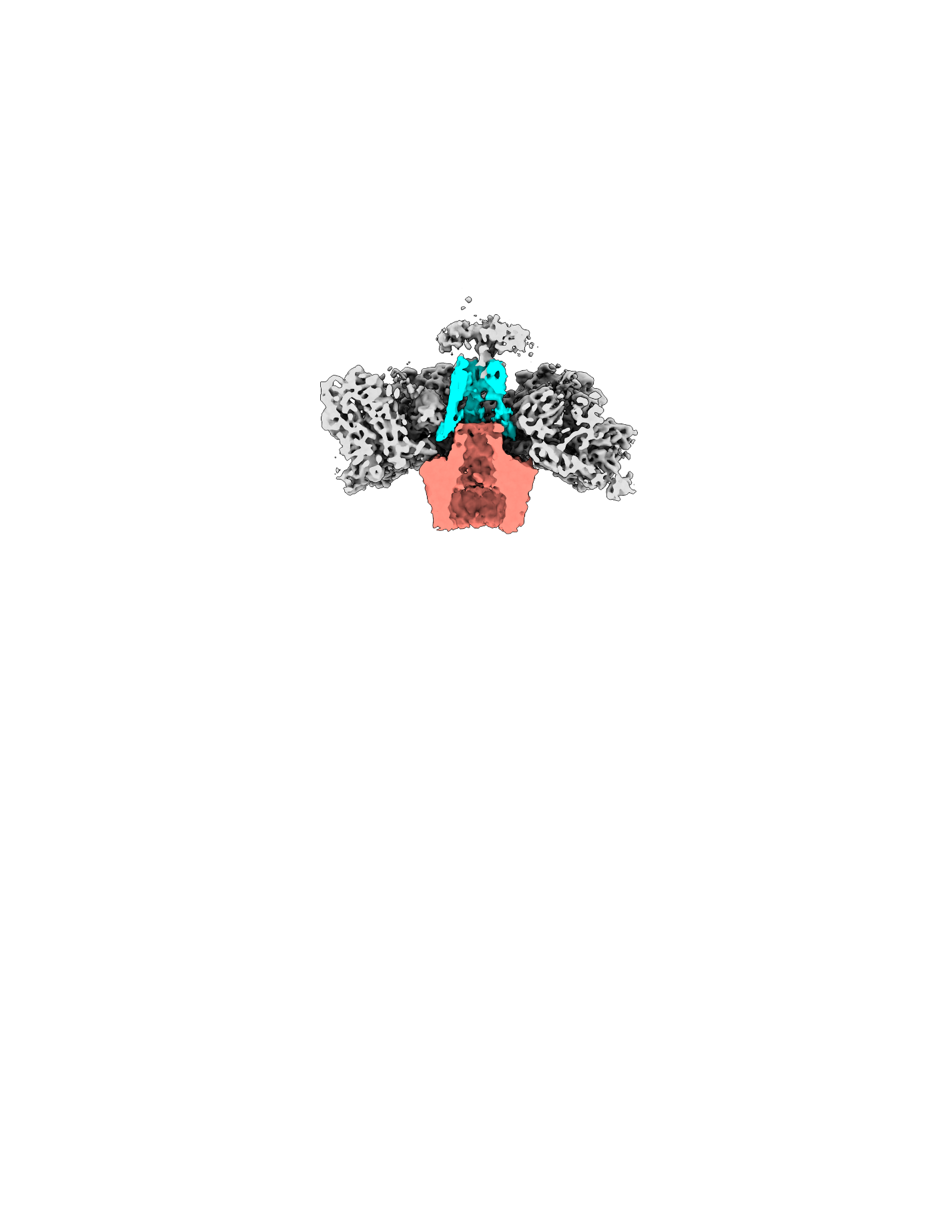


**Figure S3: Cross section of D-capsid portal vertex without terminal genome.**

**Table S1. CryoEM data collection, validation, and refinement statistics.**

|  | WT HSV-1 A whole capsid  EMDB: EMD-70689 | WT HSV-1 B whole capsid  EMDB: EMD-70690 | WT HSV-1 C Whole capsid  EMDB: EMD-70693 | WT HSV-1 D whole capsid  EMDB: EMD-70694 |
| --- | --- | --- | --- | --- |
| **Data collection** |  |  |  |  |
| Magnification | 81,000 | 81,000 | 81,000 | 81,000 |
| Voltage (kV) | 300 | 300 | 300 | 300 |
| Electron exposure (e^-^/Å^2^) | 45 | 45 | 45 | 45 |
| Defocus range (μm) | -1.5 to -2.5 | -1.5 to -2.5 | -1.5 to -2.5 | -1.5 to -2.5 |
| Pixel size (Å) | 1.1 | 1.1 | 1.1 | 1.1 |
| Symmetry imposed | C1 | C1 | C1 | C1 |
| Final particle images | 17,168 | 42,733 | 12,839 | 22,983 |
| Map resolution (Å) | 9.2 | 9.1 | 8.9 | 8.9 |
| FSC threshold | 0.143 | 0.143 | 0.143 | 0.143 |
| **Refinement** |  |  |  |  |
| Initial model used (PDB) | N/A | N/A | N/A | N/A |
| Model resolution (Å) | N/A | N/A | N/A | N/A |
| FSC threshold | N/A | N/A | N/A | N/A |
| Map sharpening B factor (Å^2^) | N/A | N/A | N/A | N/A |
| No. non-hydrogen atoms | N/A | N/A | N/A | N/A |
| Protein residues | N/A | N/A | N/A | N/A |
| *B*-factors |  |  |  |  |
| Protein | N/A | N/A | N/A | N/A |
| R.m.s. deviations |  |  |  |  |
| Bond lengths (Å) | N/A | N/A | N/A | N/A |
| Bond angles (°) | N/A | N/A | N/A | N/A |
| Validation |  |  |  |  |
| MolProbity score | N/A | N/A | N/A | N/A |
| Clash score | N/A | N/A | N/A | N/A |
| Poor rotamers (%) | N/A | N/A | N/A | N/A |
| Ramachandran plot |  |  |  |  |
| Favored (%) | N/A | N/A | N/A | N/A |
| Allowed (%) | N/A | N/A | N/A | N/A |
| Disallowed (%) | N/A | N/A | N/A | N/A |

**Table S1. CryoEM data collection, validation, and refinement statistics.**

|  | WT HSV-1 A capsid C1 portal vertex  EMDB: EMD-70699 | WT HSV-1 B capsid C1 portal vertex  EMDB: EMD-70688 | WT HSV-1 C capsid C1 portal vertex  EMDB: EMD-70692 | WT HSV-1 D capsid C1 portal vertex  EMDB: EMD-70698 |
| --- | --- | --- | --- | --- |
| **Data collection** |  |  |  |  |
| Magnification | 81,000 | 81,000 | 81,000 | 81,000 |
| Voltage (kV) | 300 | 300 | 300 | 300 |
| Electron exposure (e^-^/Å^2^) | 45 | 45 | 45 | 45 |
| Defocus range (μm) | -1.5 to -2.5 | -1.5 to -2.5 | -1.5 to -2.5 | -1.5 to -2.5 |
| Pixel size (Å) | 1.1 | 1.1 | 1.1 | 1.1 |
| Symmetry imposed | C1 | C1 | C1 | C1 |
| Final particle images | 72,728 | 66,591 | 19,345 | 135,916 |
| Map resolution (Å) | 4.4 | 4.0 | 4.2 | 4.1 |
| FSC threshold | 0.143 | 0.143 | 0.143 | 0.143 |
| **Refinement** |  |  |  |  |
| Initial model used (PDB) | N/A | N/A | N/A | N/A |
| Model resolution (Å) | N/A | N/A | N/A | N/A |
| FSC threshold | N/A | N/A | N/A | N/A |
| Map sharpening B factor (Å^2^) | -80 | -102 | -37 | -92 |
| No. non-hydrogen atoms | N/A | N/A | N/A | N/A |
| Protein residues | N/A | N/A | N/A | N/A |
| *B*-factors |  |  |  |  |
| Protein | N/A | N/A | N/A | N/A |
| R.m.s. deviations |  |  |  |  |
| Bond lengths (Å) | N/A | N/A | N/A | N/A |
| Bond angles (°) | N/A | N/A | N/A | N/A |
| Validation |  |  |  |  |
| MolProbity score | N/A | N/A | N/A | N/A |
| Clash score | N/A | N/A | N/A | N/A |
| Poor rotamers (%) | N/A | N/A | N/A | N/A |
| Ramachandran plot |  |  |  |  |
| Favored (%) | N/A | N/A | N/A | N/A |
| Allowed (%) | N/A | N/A | N/A | N/A |
| Disallowed (%) | N/A | N/A | N/A | N/A |

**Table S1. CryoEM data collection, validation, and refinement statistics.**

|  | WT HSV-1 A capsid C5 portal vertex  EMDB: EMD-70683 | WT HSV-1 B capsid C5 portal vertex  EMDB: EMD-70684 | WT HSV-1 C capsid C5 portal vertex  EMDB: EMD-70725  PDB (portal turrets): 9OPV | WT HSV-1 D capsid C5 portal vertex  EMDB: EMD-70687  PDB (portal turrets): 9OPB |
| --- | --- | --- | --- | --- |
| **Data collection** |  |  |  |  |
| Magnification | 81,000 | 81,000 | 81,000 | 81,000 |
| Voltage (kV) | 300 | 300 | 300 | 300 |
| Electron exposure (e^-^/Å^2^) | 45 | 45 | 45 | 45 |
| Defocus range (μm) | -1.5 to -2.5 | -1.5 to -2.5 | -1.5 to -2.5 | -1.5 to -2.5 |
| Pixel size (Å) | 1.1 | 1.1 | 1.1 | 1.1 |
| Symmetry imposed | C5 | C5 | C5 | C5 |
| Final particle images | 30,298 | 66,591 | 252,150 | 135,916 |
| Map resolution (Å) | 3.9 | 3.7 | 3.4 | 3.5 |
| FSC threshold | 0.143 | 0.143 | 0.143 | 0.143 |
| **Refinement** |  |  |  |  |
| Initial model used (PDB) | N/A | N/A | N/A | N/A |
| Model resolution (Å) | N/A | N/A | 3.9 | 3.7 |
| FSC threshold | N/A | N/A | 0.5 | 0.5 |
| Map sharpening B factor (Å^2^) | -94.5 | -102 | -127 | -112 |
| No. non-hydrogen atoms | N/A | N/A | 6145 | 6145 |
| Protein residues | N/A | N/A | 780 | 780 |
| *B*-factors |  |  |  |  |
| Protein | N/A | N/A | 119.55 | 99.96 |
| R.m.s. deviations |  |  |  |  |
| Bond lengths (Å) | N/A | N/A | 0.004 | 0.004 |
| Bond angles (°) | N/A | N/A | 0.861 | 0.803 |
| Validation |  |  |  |  |
| MolProbity score | N/A | N/A | 1.46 | 1.38 |
| Clash score | N/A | N/A | 8.63 | 6.92 |
| Poor rotamers (%) | N/A | N/A | 0.00 | 0.16 |
| Ramachandran plot |  |  |  |  |
| Favored (%) | N/A | N/A | 100.00 | 100.00 |
| Allowed (%) | N/A | N/A | 0.00 | 0.00 |
| Disallowed (%) | N/A | N/A | 0.00 | 0.00 |

**Table S1. CryoEM data collection, validation, and refinement statistics.**

|  | WT HSV-1 A capsid C12 portal vertex  EMDB: EMD-70678  PDB (portal dodecamer): 9OP4 | WT HSV-1 B capsid C12 portal vertex  EMDB: EMD-70679  PDB (portal dodecamer): 9OP5 | WT HSV-1 C capsid C12 portal vertex  EMDB: EMD-70691  PDB (portal dodecamer): 9OPC | WT HSV-1 D capsid C12 portal vertex  EMDB: EMD-70682  PDB (portal dodecamer): 9OP8 |
| --- | --- | --- | --- | --- |
| **Data collection** |  |  |  |  |
| Magnification | 81,000 | 81,000 | 81,000 | 81,000 |
| Voltage (kV) | 300 | 300 | 300 | 300 |
| Electron exposure (e^-^/Å^2^) | 45 | 45 | 45 | 45 |
| Defocus range (μm) | -1.5 to -2.5 | -1.5 to -2.5 | -1.5 to -2.5 | -1.5 to -2.5 |
| Pixel size (Å) | 1.1 | 1.1 | 1.1 | 1.1 |
| Symmetry imposed | C12 | C12 | C12 | C12 |
| Final particle images | 363,576 | 799,092 | 1,863,132 | 1,630,992 |
| Map resolution (Å) | 3.6 | 3.5 | 3.8 | 3.8 |
| FSC threshold | 0.143 | 0.143 | 0.143 | 0.143 |
| **Refinement** |  |  |  |  |
| Initial model used (PDB) | 6OD7 | 6OD7 | 6OD7 | 6OD7 |
| Model resolution (Å) | 3.8 | 3.6 | 3.9 | 3.9 |
| FSC threshold | 0.5 | 0.5 | 0.5 | 0.5 |
| Map sharpening B factor (Å^2^) | -137.5 | -154.5 | -191.9 | -179.1 |
| No. non-hydrogen atoms | 40512 | 40512 | 39660 | 39660 |
| Protein residues | 5076 | 5076 | 4968 | 4968 |
| Mean *B*-factors |  |  |  |  |
| Protein | 66.58 | 53.11 | 115.26 | 79.80 |
| R.m.s. deviations |  |  |  |  |
| Bond lengths (Å) | 0.004 | 0.004 | 0.004 | 0.004 |
| Bond angles (°) | 0.941 | 0.937 | 0.932 | 0.907 |
| Validation |  |  |  |  |
| MolProbity score | 1.00 | 1.28 | 1.36 | 1.21 |
| Clash score | 1.77 | 3.79 | 4.88 | 3.67 |
| Poor rotamers (%) | 0.00 | 0.02 | 0.00 | 0.00 |
| Ramachandran plot |  |  |  |  |
| Favored (%) | 97.70 | 97.42 | 97.48 | 97.78 |
| Allowed (%) | 2.30 | 2.58 | 2.52 | 2.22 |
| Disallowed (%) | 0.00 | 0.00 | 0.00 | 0.00 |

**Table S1. CryoEM data collection, validation, and refinement statistics.**

|  | WT HSV-1 B whole capsid with global scaffold  EMDB: EMD-70695 |
| --- | --- |
| **Data collection** |  |
| Magnification | 81,000 |
| Voltage (kV) | 300 |
| Electron exposure (e^-^/Å^2^) | 45 |
| Defocus range (μm) | -1.5 to -2.5 |
| Pixel size (Å) | 1.1 |
| Symmetry imposed | C1 |
| Final particle images | 18085 |
| Map resolution (Å) | 8.9 |
| FSC threshold | 0.143 |
| **Refinement** |  |
| Initial model used (PDB) | N/A |
| Model resolution (Å) | N/A |
| FSC threshold | N/A |
| Map sharpening B factor (Å^2^) | -745 |
| No. non-hydrogen atoms | N/A |
| Protein residues | N/A |
| *B*-factors |  |
| Protein | N/A |
| R.m.s. deviations |  |
| Bond lengths (Å) | N/A |
| Bond angles (°) | N/A |
| Validation |  |
| MolProbity score | N/A |
| Clash score | N/A |
| Poor rotamers (%) | N/A |
| Ramachandran plot |  |
| Favored (%) | N/A |
| Allowed (%) | N/A |
| Disallowed (%) | N/A |

**Movie S1.** 3DFlex volume series of the A-capsid portal vertex, showing slight sway of the basket relative to the capsid shell, as well as substantial variability in height.

**Movie S2.** 3DFlex volume series of the A-capsid floor at the portal vertex, showing rotation of portal relative to the capsid shell.

**Movie S3.** 3DFlex volume series of the B-capsid portal vertex, showing sway of the basket relative to the capsid shell.

**Movie S4.** 3DFlex volume series of the B-capsid floor at the portal vertex, showing rotation of portal relative to the capsid shell.

**Movie S5.** 3DFlex volume series of the C-capsid portal vertex, showing sway of the basket relative to the capsid shell.

**Movie S6.** 3DFlex volume series of the C-capsid floor at the portal vertex, showing rotation of portal relative to the capsid shell.

**Movie S7.** 3DFlex volume series of the D-capsid portal vertex, showing sway of the basket relative to the capsid shell.

**Movie S8.** 3DFlex volume series of the D-capsid floor at the portal vertex, showing rotation of portal relative to the capsid shell.
